# Thyroid eye disease in paediatric Graves’ disease: a case series from the Gulf region with comparison to adults

**DOI:** 10.1007/s10792-026-04128-1

**Published:** 2026-06-12

**Authors:** Fatema Mohamed Ali Abdulla Aljufairi, Kenneth Ka Hei Lai, Sayed Fadhel Hashem Jaafar Neama, Fatema Hani Abdulla Saleh, Khadija Ali Alola, Fayza Ahmed Al-jenaidi, Mansoor Hameed Rajab, Maha Mahmood Alarayedh, Wadeea Fareed Mohamed, Ebrahim Hassan Matar, Wafa Fawzi Hassan, Adel Salman Alsayyad, Clement Chee Yung Tham, Chi Pui Pang, Kelvin Kam Lung Chong

**Affiliations:** 1 Department of Ophthalmology and Visual Sciences, Faculty of Medicine, The Chinese University of Hong Kong, Hong Kong Eye Hospital, 147K Argyle Street, Kowloon, Hong Kong Special Administrative Region China; 2 Department of Ophthalmology, Salmaniya Medical Complex, Government Hospitals, Manama, Bahrain; 3 Almanal Eye Hospital, Manama, Bahrain; 4 Department of Ophthalmology and Visual Sciences, Prince of Wales Hospital, Sha Tin, Hong Kong Special Administrative Region China; 5 Eastern Health Cluster, Dammam, Saudi Arabia; 6 American Mission Hospital, Manama, Bahrain; 7 Ibn Sina Medical Center, Manama, Bahrain; 8 Directorate of Public Health, Ministry of Health, Manama, Bahrain; 9 Department of Family and Community Medicine, College of Medicine and Medical Sciences, Arabian Gulf University, Manama, Bahrain; 10 Hong Kong Eye Hospital, Kowloon, Hong Kong Special Administrative Region China; 11 Eye Centre, The Chinese University of Hong Kong Medical Centre, Sha Tin, Hong Kong Special Administrative Region China; 12 Department of paediatrics, Arabian Gulf University, Manama, Bahrain; 13 Department of paediatrics, King Hamad University Hospital, Muharraq, Bahrain

## Abstract

**Purpose:**

To characterize the demographic characteristics, clinical features, and severity of thyroid eye disease (TED) in paediatric patients with Graves’ disease for comparison with an adult cohort in the Kingdom of Bahrain.

**Methods:**

Paediatric patients (≤ 18 years) with Graves’ disease were screened for TED in Bahrain between October and December 2025. Paediatric endocrinologists across government, military, university, and private sectors participated. Patients were identified from physician-provided lists and verified using diagnostic codes and medical records from four nationwide centres. Comprehensive ophthalmic and orbital examinations were performed, and patients were classified into TED and non-TED groups. TED was diagnosed using the Bartley criteria and graded according to the European Group on Graves’ Orbitopathy (EUGOGO) classification. Findings were compared with an adult Graves’ disease cohort (> 18 years) from the same population referred between September 2023 and December 2025.

**Results:**

Among 22 paediatric patients with Graves’ disease (median age 14 years, interquartile range [IQR] 10–16 years; female-to-male ratio 4:1), 11 (50%) were diagnosed with TED. No statistically significant differences were observed between patients with and without TED regarding demographic characteristics, smoking exposure, Graves’ disease duration, family history of autoimmune thyroid disease, or hyperthyroidism treatment. However, parental consanguinity was significantly more common among patients with TED.

Among paediatric TED patients, 54.5% had bilateral disease and 81.8% had mild TED. None had sight-threatening disease or diplopia. All patients were symptomatic, most commonly with periorbital bulging or swelling (81.8%). Lid retraction (72.7%) and proptosis (63.6%) were the most frequent objective findings, whereas extraocular motility restriction was uncommon (18.2%).

Compared with adults (n = 81), mild disease was more frequent in paediatric patients (81.8% vs 48.1%; *P* = 0.04), whereas lid lag was more common in adults (64.2% vs 18.2%; *P* = 0.01).

**Conclusion:**

This study provides the first regional data on paediatric TED in the Gulf region, demonstrating that TED is common among children with Graves’ disease and is predominantly mild in severity. Eyelid involvement and proptosis were the most frequent manifestations. Parental consanguinity was more common among children with TED, suggesting a possible genetic contribution. These findings emphasize the importance of routine ophthalmic screening in paediatric Graves’ disease and highlight age-related differences in clinical presentation.

**Supplementary Information:**

The online version contains supplementary material available at 10.1007/s10792-026-04128-1.

## Introduction

Thyroid eye disease (TED), the most common extrathyroidal manifestation of Graves’ disease (GD), is an autoimmune inflammatory disorder of the orbit that can result in significant functional and psychosocial morbidity [1, 2]. The disease is driven by antibodies against the thyroid-stimulating hormone receptor, leading to orbital fibroblast activation, inflammation, and tissue expansion from glycosaminoglycan accumulation within orbital fat and extraocular muscles [1, 3–5]. While GD is uncommon in childhood, TED can occur and requires attention due to its potential impact on vision, appearance, and quality of life [6].

Paediatric TED is known to be rare, with an estimated annual incidence of 1.7–3.5 per 100,000 children [7, 8]. However, reported prevalence among children with GD varies widely [8–11]. The condition predominantly affects adolescents and females and is generally milder than adult disease [6, 7, 12], with most cases managed conservatively [9, 10]. The biological basis for age-related differences in disease severity remains incompletely understood, [13] although environmental and genetic factors, including cigarette smoke exposure and familial autoimmune thyroid disease, have been implicated [7, 14].

Despite these observations, paediatric TED has not been systematically studied in the Gulf region, and regional data on disease phenotype and severity are lacking. This study characterizes the demographic and clinical features of paediatric TED in patients with GD in Bahrain and directly compares these findings with an adult cohort from the same population. By providing the first regional analysis of paediatric TED from the Gulf, this work addresses a critical knowledge gap and offers data that may inform screening practices and clinical management in this understudied population.

## Methods

This prospective case series was conducted in the Kingdom of Bahrain between October and December 2025. All paediatric patients (≤ 18 years) with Graves’ disease were systematically screened for TED. To ensure comprehensive case ascertainment, all paediatric endocrinologists practicing across government, military, university, and private healthcare sectors were invited to participate. Recruitments were conducted across four institutions providing nationwide coverage: Salmaniya Medical Complex, King Hamad University Hospital, American Mission Hospital, and Ibn Sina Medical Centre. Eligible patients were identified from paediatric endocrinology clinic databases and hospital electronic medical records. In Bahrain, paediatric Graves’ disease is managed exclusively by paediatric endocrinologists.

All identified patients were invited for a standardized ophthalmic assessment at Salmaniya Medical Complex. TED was diagnosed according to the Bartley and Gorman criteria, [15] and patients were classified as TED or non-TED based on findings from a standardized examination performed by a single orbital and oculoplastic surgeon.

Ophthalmic evaluation included best-corrected visual acuity, refraction, intraocular pressure measurement, slit-lamp biomicroscopy, assessment of eyelid position, extraocular motility testing in all cardinal positions of gaze, cover testing for manifest strabismus, corneal exposure evaluation, colour vision testing using Ishihara plates, and optic nerve assessment. Proptosis was measured using Hertel exophthalmometry with a standardized technique and defined as values exceeding age-adjusted normative limits or inter-eye asymmetry ≥ 2 mm [16]. Upper eyelid retraction was defined as the upper eyelid margin at or above the superior corneal limbus in primary gaze.

Disease severity was classified according to the European Group on Graves’ Orbitopathy (EUGOGO) severity classification, and clinical activity was assessed using the Clinical Activity Score (CAS) [17]. Thyroid function tests (TFT) and thyroid-specific antibodies were recorded when available.

Clinical and demographic data from an adult Graves’ disease cohort (> 18 years) from the same population were included for comparison. Adult patients represented a referral-based cohort evaluated between September 2023 and December 2025, whereas paediatric patients were identified through systematic screening; therefore, comparisons were interpreted in the context of differing sampling methods.

The study adhered to the principles of the Declaration of Helsinki and received ethical approval from government hospitals (Approval No. 101–150924 and No. 112–271,125). Written informed consent was obtained from all participants and their parents or legal guardians.

Continuous variables are presented as median (interquartile range- IQR) and categorical variables as number (%). Statistical comparisons were performed using the Mann–Whitney U test for continuous variables and Fisher’s exact test for categorical variables, as appropriate. All tests were two-sided, with p < 0.05 considered statistically significant. Statistical analyses were conducted using SPSS software.

## Results

A total of 22 paediatric patients with Graves’ disease were screened, including 18 females (81.8%) and 4 males (18.2%). The median age of the study population was 14 years (IQR 10–16). TED was identified in 11 of 22 patients, corresponding to a prevalence of 50.0% (95% CI, 28.2–71.8) Table 1.

**Table 1:** Baseline characteristics of paediatric patients with Graves’ disease

Variable	All patients (n = 22)	TED (n = 11)	No TED (n = 11)	*P*-value
Age, median (IQR)	14 (10–16)	15 (11–16)	12 (8–16)	0.36^a^
Female sex, n (%)	18 (81.8)	9 (81.8)	9 (81.8)	1.00^b^
Smoking exposure^c^, n (%)	10 (45.5)	5 (45.5)	5 (45.5)	1.00^b^
Graves’ disease duration, months, median (IQR)	23 (6–43)	24 (5–72)	22 (12–43)	0.83^a^
*Family history of AITD, n (%)*	19 (86.4)	8 (72.7)	11 (100)	0.22^b^
1st degree relatives, n (%)	8 (36.4)	2 (18.2)	6 (54.5)	0.39^b^
2nd degree relatives, n (%)	11(50.0)	6 (54.5)	5 (45.5)	–
Consanguinity, n (%)	9 (47.4)	7 (87.5)	2 (18.2)	0.005^b^
*Hyperthyroidism treatment*				
Ever ATD use, n (%)	19 (86.4)	11 (100)	8 (72.7)	0.21^b^
Radioiodine therapy, n (%)	1(4.5)	1(9.1)	0 (0)	–
Thyroidectomy, n (%)	0 (0)	0 (0)	0 (0)	–

*Abbreviations*: TED, Thyroid eye disease; IQR, Inter-quartile range; n, number; AITD, Autoimmune thyroid disease; ATD, Anti thyroid drug; *P Value is significant (< 0.05)

^*a*^*P*-value *based* on Mann–Whitney U test

^*b*^*P*-value *based* on Fisher’s exact test

^*c*^Environmental tobacco exposure

Patients with TED had a median age of 15 years (IQR 11–16) compared with 12 years (IQR 8–16) in those without TED, with no statistically significant difference between groups (*P* = 0.36). Female sex predominated in both groups (81.8%; *P* = 1.00), and exposure to tobacco smoke was also similar (45.5% in each group; *P* = 1.00) Table 1.

The median duration of Graves’ disease was 24 months (IQR 5–72) in patients with TED and 22 months (IQR 12–43) in those without TED (*P* = 0.83). A family history of autoimmune thyroid disease was common and did not differ significantly between groups (72.7% vs 100%; *P* = 0.22). In contrast, parental consanguinity was significantly more frequent among patients with TED compared with those without TED (87.5% vs 18.2%; *P* = 0.005) Table 1.

Antithyroid drug (ATDs) use was observed in all patients with TED and in 72.7% of patients without TED, without a statistically significant difference (*P* = 0.21). Only one patient had received radioactive iodine (RAI) therapy, and this patient belonged to the TED group. No patients had undergone thyroidectomy Table 1.

Among patients with TED, involvement was bilateral in 6 (54.5%) and unilateral in 5 (45.5%). All affected patients were symptomatic, with the most frequently reported complaint being periorbital bulging or swelling (81.8%). Other symptoms included tearing (36.4%), blurred vision (27.3%), and foreign body sensation (27.3%), while no diplopia was reported Table 2.

**Table 2:** Clinical characteristics of thyroid eye disease (n = 11)

Characteristic	n (%)
*Laterality*	
Bilateral	6 (54.5)
Unilateral	5 (45.5)
*Presenting symptoms*	
No ocular complaints	0 (0)
Periorbital bulging/swelling	9 (81.8)
Tearing	4 (36.4)
Diplopia	0 (0)
Blurred vision	3 (27.3)
Foreign body sensation	3 (27.3)
*Eyelid signs*	
Lid retraction	8 (72.7)
Lid lag	2 (18.2)
Lagophthalmos	3 (27.3)
*Orbital and motility signs*	
Proptosis	7 (63.6)
Extraocular motility restriction	2 (18.2)
*Activity*	
Inactive (CAS < 3)	11 (100.0)
Active (CAS ≥ 3)	0 (0)
*Severity*	
Mild	9 (81.8)
Moderate-to-severe	2 (18.2)
Sight-threatening	0 (0)

Percentages calculated using the number of patients with thyroid eye disease (n = 11). Patients may have reported more than one symptom

The most frequent objective findings were lid retraction (72.7%) and proptosis (63.6%), followed by lagophthalmos (27.3%) and extraocular motility restriction (18.2%). No cases of dysthyroid optic neuropathy (DON) were observed. Disease severity was predominantly mild (81.8%), with no sight-threatening cases, and all patients were classified as inactive at presentation Table 2. Detailed orbital measurements are provided in Supplementary Table S1**.**

Laboratory data were available for a subset of patients. Abnormal TFT and thyroid antibody positivity were common and did not differ significantly between patients with and without TED. Suppressed thyroid-stimulating hormone (TSH) was observed in 54.5% of patients with TED and 63.6% of those without TED, while elevated free T4 was present in 36.4% of patients in both groups (all *P* = 1.00). Table 3.

**Table 3:** Laboratory characteristics of thyroid eye disease in paediatric and Graves’ patients

Variable	All patients (n = 22)	TED (n = 11)	No TED (n = 11)	*P*-value
*Thyroid function tests*				
Suppressed TSH, n (%)	13/22 (59.1%)	6/11 (54.5%)	7/11 (63.6%)	1.00
Elevated Free T4, n (%)	8/22 (36.4%)	4/11 (36.4%)	4/11 (36.4%)	1.00
*Thyroid antibodies*				
TRAb positive, n (%)	4/5 (80%)	2/2 (100.0%)	2/3 (66.7%)	1.00
Anti-TPO positive, n (%)	6/8 (75%)	2/3 (66.7%)	4/5 (80%)	1.00
Anti-thyroglobulin positive, n (%)	2/8 (25%)	1/4 (25%)	1/4 (25%)	1.00

*Abbreviations*: TED, Thyroid eye disease; TRAb, Thyrotropin receptor antibodies; Anti-TPO, Anti-*thyroid* peroxidase

^*^P-value is *significant* (< 0.05)

*P*-value based on Fisher’s exact test

Thyroid receptor antibody (TRAb) positivity was detected in 100.0% of tested patients with TED compared with 66.7% of those without TED, anti-thyroid peroxidase (anti-TPO) positivity in 66.7% and 80.0%, respectively, and anti-thyroglobulin positivity in 25.0% of both groups (all *P* = 1.00).Table 3.

In comparison with adults (n = 81), no statistically significant differences were observed in sex distribution, smoking exposure, or overall family history of autoimmune thyroid disease. However, a second-degree family history of autoimmune thyroid disease was more frequent among paediatric patients (54.5% vs 8.6%; *P* = 0.001) Table 4. ATDs use was universal among paediatric patients and was less frequent in adults (100% vs 76.5%; *P* = 0.04). The use of RAI therapy did not differ statistically between groups (9.1% vs 29.6%; *P* = 0.17), and no paediatric patients had undergone thyroidectomy compared with 9.9% of adults (*P* = 0.57) Table 4.

**Table 4:** Baseline characteristics of paediatric and adult patients with Graves’ disease

Variable	Paediatric n (%)	Adult n (%)	*P*-value
Number of patients	11	81	–
Age, median (IQR)	15 (11–16)	44 (33–54)	** < 0.001**^a^
Female sex, n (%)	9 (81.8)	56 (69.1)	0.54^b^
Smoking exposure, n (%)	5 (45.5)	38 (46.9)	1.00^b^
*Family history of AITD, n (%)*	8 (72.7)	52 (64.2)	0.74^b^
1st degree relatives, n (%)	2 (18.2)	36 (44.4)	0.12^b^
2nd degree relatives, n (%)	6 (54.5)	7 (8.6)	**0.001**^b^
3rd degree relatives, n (%)	0 (0)	9 (11.1)	0.58^b^
*Hyperthyroidism treatment*			
Ever ATD use, n (%)	11 (100)	62 (76.5)	**0.04**^b^
Radioiodine therapy, n (%)	1(9.1)	24 (29.6)	0.17^b^
Thyroidectomy, n (%)	0 (0)	8 (9.9)	0.57^b^

*Abbreviations*: TED, Thyroid eye disease; IQR, Inter-quartile range; n, number; AITD, Autoimmune thyroid disease; ATD, Anti thyroid drug; * p-value is significant (< 0.05)

^*a*^*P*-value based on Mann–Whitney U test

^*b*^*P*-value based on Fisher’s exact test

In terms of clinical features, paediatric patients more frequently exhibited mild disease (81.8% vs 48.1%; *P* = 0.04), whereas lid lag was more common in adults (64.2% vs 18.2%; *P* = 0.01). No statistically significant differences were observed between paediatric and adult patients in the prevalence of proptosis, lagophthalmos, extraocular motility (EOM) restriction, or diplopia Table 5.

**Table 5:** Clinical characteristics of thyroid eye disease in paediatric and adult patients

Characteristic	Paediatric n (%)	Adult n (%)	*p*-value
Number of patients	11	81	–
*Laterality*			
Bilateral	6 (54.5)	54 (66.7)	0.50
Unilateral	5 (45.5)	27 (33.3)	–
*Eyelid signs*			
Lid retraction	8 (72.7)	52 (64.2)	0.71
Lid lag	2 (18.2)	52 (64.2)	0.01*
Lagophthalmos	3 (27.3)	37 (45.7)	0.33
*Orbital and motility signs*			
Proptosis	7 (63.6)	55 (67.9)	0.78
Extraocular motility restriction	2 (18.2)	32 (39.5)	0.32
Diplopia	0 (0)	9 (11.1)	0.34
*Activity*			
Inactive (CAS < 3)	11 (100)	78 (96.3)	1.00
Active (CAS ≥ 3)	0 (0)	3 (3.7)	–
*Severity*			
Mild	9 (81.8)	39 (48.1)	0.04*
Moderate-to-severe	2 (18.2)	42 (51.9)	–
Sight-threatening	0 (0)	0 (0)	–

^*^P-*value* is significant (< 0.05)

*P*-*value* based on Fisher’s exact test

## Discussion

In this nationwide paediatric cohort, TED was identified in half of children with GD, highlighting the substantial burden of ocular involvement. TED occurred predominantly in adolescent females and was not associated with differences in age, disease duration, environmental tobacco exposure, family history of autoimmune thyroid disease, or biochemical thyroid status. However, parental consanguinity was significantly more common among children with TED than those without TED. Clinical presentation was largely mild and inactive, with lid retraction and proptosis representing the most frequent objective findings. Notably, no cases of dysthyroid optic neuropathy or sight-threatening disease were observed.

Compared with adults from the same population, the paediatric patients demonstrated a milder disease phenotype, reflected by a higher proportion classified as mild and a lower prevalence of lid lag. While overall demographic characteristics were similar between age groups, a second-degree family history of autoimmune thyroid disease was more frequent in children, suggesting possible age-related differences in genetic or familial susceptibility despite shared autoimmune pathophysiology.

This study has several limitations. The sample size was small given the rarity of paediatric Graves’ disease within a defined national population, which limited the study power to detect statistical difference between the 2groups. However, systematic nationwide case ascertainment through direct engagement with all paediatric endocrinologists across healthcare sectors reduces selection bias and enhances representativeness. Additionally, while the paediatric cohort was prospectively screened, the adult comparison group was referral-based, limiting direct comparison. To address this, adult analyses were interpreted descriptively in light of the differing recruitment pathways. Biochemical data were incomplete for a subset of patients, particularly TRAb testing, which was limited by laboratory availability and less frequent clinical utilization in patients with overt biochemical hyperthyroidism. This may have reduced the ability to fully assess serological associations. Nevertheless, the primary outcomes, including clinical phenotype and disease severity, were evaluated using standardized diagnostic criteria and uniform ophthalmic examination. Although assessment by a single orbital specialist may limit generalizability, it ensured methodological consistency and minimized interobserver variability.

To our knowledge, this study represents the first nationwide characterization of paediatric TED in the Gulf region and one of the few to directly compare paediatric and adult disease within a shared population. The use of standardized diagnostic criteria, uniform assessment, and established severity classifications ensured consistent phenotyping. By achieving near-complete nationwide case identification within a specialist-managed healthcare system, this study provides population-level insight rather than clinic-based estimates.

In addition, this study is among the first to explore parental consanguinity as a potential factor associated with paediatric TED in a Gulf population, where consanguineous marriage remains relatively common.

Our findings are broadly consistent with patterns identified in published paediatric studies across Western, [9, 10, 18–22] Asian [11, 13, 23–26], and Middle Eastern populations [27]. Across populations, paediatric TED consistently demonstrates female predominance [9–11, 13, 18–28], predominantly mild disease severity, [11, 23, 24, 27] and absence of dysthyroid optic neuropathy [9, 11, 13, 18, 19, 22–24, 26]. The predominance of lid retraction [10, 19, 22, 24, 25, 28] and proptosis [13, 18, 19, 21, 23, 24, 28] observed in our cohort mirrors the most frequently reported clinical signs internationally. However, bilateral involvement in our cohort was lower than rates reported in Southeast Asian, [23] East Asian, [13] and Western cohorts, [18] suggesting potential regional variation in orbital expression. Furthermore, while extraocular motility restriction fell within reported international ranges, [20–24] the absence of diplopia contrasts with some Western series, [20, 28] highlighting possible population-level differences in functional impact.

In comparison with the previously published Chinese cohort by Lai KKH et al. [13], the overall pattern of milder paediatric disease relative to adults was consistent. Both studies demonstrated absence of optic neuropathy and lower disease severity in children. However, the higher rate of bilateral involvement reported in the Chinese population, compared with the more balanced laterality observed in our Gulf cohort, suggests potential ethnic or environmental influences on orbital expression. Importantly, unlike prior clinic-based investigations, the present study employed systematic nationwide screening, enabling estimation of TED prevalence within a defined paediatric Graves’ disease population.

Compared with international cohorts, management patterns in our population were consistent with the predominantly conservative therapeutic approach reported in paediatric TED. ATDs represented the principal treatment modality in our cohort, paralleling the high rates reported in East and Southeast Asian studies, where ATD use approaches universal first-line therapy, [11, 24] while Western cohorts demonstrate greater variability in treatment strategies [18, 19, 22] In contrast, radioactive iodine therapy and thyroidectomy were uncommon in our paediatric population, mirroring trends observed across Asian cohorts, [11, 13, 23, 26] but differing from several Western studies where definitive therapies are used more frequently [18, 19, 22, 29, 30] Similarly, no patients in our cohort required systemic corticosteroids, orbital decompression, or other aggressive ophthalmic interventions, consistent with the predominantly mild and inactive phenotype observed in our cohort. Whether these differences reflect regional therapeutic preferences, referral patterns, or intrinsic biological variation remains uncertain.

Collectively, these findings reinforce the concept that paediatric TED represents a distinct, generally milder clinical phenotype across ethnic groups, while suggesting that specific features such as laterality and functional ocular manifestations may vary regionally.

These observations also define important directions for future research. Larger multicentre or multinational collaborative studies are required to validate prevalence estimates and clarify regional variation in clinical expression. Standardized prospective methodologies would strengthen cross-population comparisons and reduce referral-related bias. The higher frequency of parental consanguinity and second-degree family history observed in our paediatric cohort suggests a potential role for genetic susceptibility in early-onset disease and warrants further genetic investigation. Longitudinal follow-up studies are particularly needed to determine whether the predominantly mild and inactive phenotype observed at presentation persists over time and to refine risk assessment and age-specific management strategies.

## Supplementary Information

Below is the link to the electronic supplementary material.

Supplementary file1 (DOCX 17 kb)

## Data Availability

No datasets were generated or analysed during the current study.

## Abbreviations

TED: Thyroid eye disease
EUGOGO: European group on Graves’ orbitopathy
GD: Graves’ disease
CAS: Clinical activity score
IQR: Interquartile range
ATDs: Anti-thyroid drugs
RAI: Radioactive iodine
DON: Dysthyroid optic neuropathy
TSH: Thyroid stimulating hormone
TRAb: Thyrotropin receptor antibodies
Anti-TPO: Anti-thyroid peroxidase antibody
TgAb: Anti-thyroglobulin antibodies
EOM: Extraocular motility
