# Supplementary material for: Thyroid eye disease in paediatric Graves’ disease: a case series from the Gulf region with comparison to adults
Source: Int Ophthalmol. 2026 Jun 12;46(1):262. doi: 10.1007/s10792-026-04128-1 (PMC13263274; doi:10.1007/s10792-026-04128-1)
Supplement: Supplementary file 1 — Supplementary file1 (DOCX 17 kb) [file 10792_2026_4128_MOESM1_ESM.docx]

| **Case no.** | **Age**  **(years)** | **Sex** | | **Severity** | | **MRD1**  **(mm)** | **MRD2**  **(mm)** | | **Lagophthalmos**  **(mm)** | | **Hertel**  **(mm)** | |  | | **Lid Lag**  **(mm)** | |
| --- | --- | --- | --- | --- | --- | --- | --- | --- | --- | --- | --- | --- | --- | --- | --- | --- |
| **1** | 8 | | F | | Mild | R 5.0, L 5.5 | | R 5.0, L 5.0 | R 0.0, L 0.0 | R 17.0, L 18.0* | |  | | R 0.0, L 0.0 | |  |
| **2** | 10 | | F | | Mild | R 5.0, L 3.0 | | R 6.0, L 6.0 | R 0.0, L 0.0 | R 16.0, L 14.0* | |  | | R 0.0, L 0.0 | |  |
| **3** | 11 | | F | | Mild | R 3.0, L 3.0 | | R 4.0, L 4.0 | R 0.0, L 0.0 | R 17.0, L 16.0 | |  | | R 1.0, L 0.5 | |  |
| **4** | 13 | | F | | Moderate to severe | R 7.0, L8.0 | | R 7.0, L8.0 | R 1.0, L 3.0 | R 18.0, L 19.5* | |  | | R 1.0, L 2.0 | |  |
| **5** | 15 | | F | | Mild | R 3.0, L 4.0 | | R 5.0, L 5.0 | R 0.0, L 0.0 | R 17.0, L 18.0 | |  | | R 0.0, L 0.0 | |  |
| **6** | 15 | | F | | Mild | R 3.5, L 3.0 | | R 6.0, L 5.0 | R 0.0, L 0.0 | R 20.0, L 19.0* | |  | | R 0.0, L 0.0 | |  |
| **7** | 15 | | M | | Mild | R 3.0, L 3.0 | | R 7.0, L 7.0 | R 0.0, L 0.0 | R 19.0, L 19.0 | |  | | R 0.0, L 0.0 | |  |
| **8** | 16 | | M | | Mild | R 4.0, L 5.0 | | R 6.0, L 6.0 | R 0.0, L 0.0 | R 19.0, L 18.0* | |  | | R 0.0, L 0.0 | |  |
| **9** | 16 | | F | | Mild | R 4.0, L 3.5 | | R 7.0, L 7.0 | R 0.0, L 0.0 | R 19.0, L 17.0* | |  | | R 0.0, L 0.0 | |  |
| **10** | 17 | | F | | Mild | R 3.5, L 3.5 | | R 7.0, L 7.0 | R 2.0, L1.0 | R 17.0, L 16.0 | |  | | R 1.0, L 0.0 | |  |
| **11** | 18 | | F | | Moderate to severe | R 5.0, L 6.0 | | R 6.0, L 7.0 | R 0.0, L 0.0 | R 19.5, L 20.5* | |  | | R 0.0, L 0.0 | |  |

**Supplementary Table S1. Detailed orbital measurements in paediatric thyroid eye disease (n = 11)**

MRD1= Margin reflex distance 1, MRD2= margin reflex distance 2, EOM= Extraocular motility, R= Right, L= Left.

Proptosis was defined as a Hertel exophthalmometry value (mean + 2 SD based on normative population data) or an inter-eye asymmetry of ≥2 mm:

Ages 6–12 years: ≥17.8 mm

Ages 13–18 years: ≥19.0 mm

Asterisk denotes values meeting criteria for proptosis
